# Alpha-1 Antitrypsin Screening in a Selected Cohort of Patients Affected by Chronic Pulmonary Diseases in Naples, Italy

**DOI:** 10.3390/jcm10081546

**Published:** 2021-04-07

**Authors:** Anna Annunziata, Ilaria Ferrarotti, Antonietta Coppola, Maurizia Lanza, Pasquale Imitazione, Sara Spinelli, Pierpaolo Di Micco, Giuseppe Fiorentino

**Affiliations:** 1 Unit of Respiratory Physiopathology, Department of Critic Area, Monaldi Hospital, 80131 Naples, Italy; antonietta.coppola84@gmail.com (A.C.); maurizia.lanza85@gmail.com (M.L.); pasqualeimitazione@gmail.com (P.I.); spinelli.sara@outlook.it (S.S.); giuseppefiorentino1@gmail.com (G.F.); 2 Center for Diagnosis of Inherited Alpha1-Antitrypsin Deficiency, Pneumology Unit, Department of Internal Medicine and Therapeutics, IRCCS San Matteo Hospital Foundation, University of Pavia, 27100 Pavia, Italy; i.ferrarotti@smatteo.pv.it; 3 Department of Medicine, Buon Consiglio Fatebenefratelli Hospital of Naples, 80128 Naples, Italy; pdimicco@libero.it

**Keywords:** alpha1 antitrypsin deficiency, chronic obstructive pulmonary disease, bronchiectasis, asthma, emphysema

## Abstract

Introduction. Alpha-1 antitrypsin deficiency (AATD) is a genetic condition associated with several respiratory diseases in patients with severe protein deficiency. AATD is often late diagnosed or underdiagnosed. Diagnosis frequently occurs in patients with chronic obstructive pulmonary disease and emphysema characterized by frequent exacerbations and over ten years’ duration. The purpose of this study was to evaluate the incidence of alpha-1 antitrypsin deficiency in patients with the chronic pulmonary disease after a thorough screening in the city of Naples in southern Italy. Materials and methods. Two hundred patients suffering from respiratory pathology (chronic obstructive pulmonary disease (COPD), emphysema, asthma, or bronchiectasis) were examined and evaluated in our outpatients’ clinic and tested for serum levels of AAT. Patients who had a respiratory disease suspected of AATD and/or serum AAT < 120 mg/dL underwent genetic testing. Genetic screening was performed on samples from 141 patients. Results. A total of 36 patients had an intermediate deficiency of AAT levels. Among them, 8 were PI*MZ, 6 were PI*MS and 22 had rare pathological mutations. Five patients had a severe AATD, all were composite heterozygous with S or Z allele, while the other allele had a rare pathological mutation. Conclusions. The incidence of genetic defects as AATD in the population of patients affected by chronic respiratory disorders is always a matter of discussion because of the frequent interaction between genes and environmental causes. In our series, numerous rare variants and compound heterozygosity have been described. No homozygous patients have been described. The present is one of few studies available on the incidence of rare variants in the geographic area of the city of Naples. So, our results could be considered interesting not only to know the incidence of AATD and its related rare mutations but also to support early diagnosis and treatments for patients with chronic pulmonary disease and frequent exacerbation and to fight the association with environmental causes of pulmonary damages as smoking.

## 1. Introduction

Alpha-1 antitrypsin (AAT) is a circulating glycoprotein, and its main function is to inhibit neutrophil elastase and other serine proteases in blood and tissues [1,2,3].

AAT is the most prevalent protease inhibitor in human serum (90–200 mg/dL), and it is also coded as SERPINA1 (serine protease inhibitor, group A, member 1) gene. AAT deficiency (AATD) was first identified in 1963 by Laurell and Eriksson. From a clinical point of view, the absence of the AAT in the alpha-1 electrophoresis band was observed in patients who had developed emphysema at a young age [4].

Epidemiologically, AATD deficiency is a relatively common genetic disorder [5,6]. In a study compiling data from 97 countries, approximately 190 million cases of AATD were estimated out of a total population of 5.2 billion or a prevalence of about 3.6% [7]. Of these 190 million estimated cases of AATD, approximately 75% will have a mild deficiency that does not increase their risk of the onset of the clinical disease. Serum protein deficiency can be severe when both alleles are pathological, or intermediate when one of the two alleles is pathological. The serological decrease may be due to genetic alteration. AATD is an autosomal codominant genetic condition that mainly affects Caucasians of European heritage due to the presence of a deficient allele that could be identified as S allele or Z allele according to the type of genetic mutation. Of the remaining 25%, almost all (24%) are heterozygous with one normal allele (M) and one deficient allele (S or Z). There are data of increased disease risk in the presence of these mutations. The SZ (0.7%) phenotype and the ZZ (0.1%) genotypes have a well-documented increase in their risk of AATD-associated diseases [7]. Furthermore, although S and Z are the most common mutations, 150 different mutations of SERPINA1 have been reported [8,9].

Currently, the World Health Organization, the American Thoracic Society, and the European Respiratory Society recommend screening for AATD in patients suffering from the chronic obstructive pulmonary disease (COPD), emphysema, bronchiectasis and asthma. The clinical course of the following respiratory diseases in homozygous patients with AATD is severe [10]. Typically, patients show an early onset of COPD and emphysema in adults and liver disease. Less frequently, AAT deficiency is associated with asthma, systemic vasculitis, neutrophilic panniculitis, and other inflammatory, autoimmune and neoplastic diseases.

Some individuals with genetic variants manifest overt clinical diseases, while others have only minor symptoms [11,12]. Often the diagnosis is made in patients with COPD and emphysema with frequent exacerbations of over 10 years duration. Of course, early diagnosis of these patients could be beneficial to improve the outcome and also to provide additional motivation for smoking cessation and avoidance of second-hand smoke, which could reduce the risk of lung damage. Diagnosis of AATD could also provide an additional avenue for treatment of lung dysfunction with AAT replacement therapy. The first step in diagnosing AATD is the measurement of AAT level in plasma or serum. This is a simple, inexpensive, and widely available test. AAT level can be determined by radial immunodiffusion, nephelometry, or turbidimetry. Currently, the preferred method is nephelometry, because radial immunodiffusion tends to overestimate the AAT concentration [13]. The reference range for serum AAT concentrations in adults is usually 90–200 mg/dL, corresponding to 20–60 µmol/dL. Sensitive, specific quantification of plasma AAT by immunoturbidimetry or nephelometry is followed by isoelectric focusing to determine the phenotypic variants and/or by genotyping. Phenotyping and genotyping are carried out by only a few specialized laboratories. Homozygous or compound heterozygous patients generally have a severely deficient serum level, <50 mg/dL; heterozygotes, depending on the different mutations, may have a serum level of alpha 1 antitrypsin, sometimes even higher than 90 mg/dL. The clinical significance of these intermediate levels of AAT has always been debated.

Yet, AATD remains underdiagnosed, despite the recommendations of international health organizations for broader screening. So, the purpose of this study is to evaluate the incidence of AATD in a cohort of 200 patients in our specialist outpatients’ clinic for pulmonary diseases in the geographic area of the city of Naples in Southern Italy.

## 2. Materials and Methods

From October 2018 to May 2019, 200 patients who were examined in our clinic (Division of Respiratory Physiopathology, Monaldi Hospital, Naples, Italy) for various respiratory diseases were also screened for serum AAT and the C-reactive protein.

The patients suffered from one of the following conditions for at least five years: COPD, emphysema, asthma, bronchiectasis of unknown etiology, or obstructive sleep apnea syndrome with persistent diurnal dyspnea.

Familial anamnesis was negative for AATD in first degree relatives, and all patients never measured their serum AAT levels before this study.

All patients quantified plasma AAT by nephelometry. Patients who had a suspected clinical picture associated with serum AAT levels <120 mg/dL with normal C-reactive protein (normal value < 1) underwent further testing. These patients performed high-resolution CT lung scan.

Furthermore, samples underwent subjected qualitative analysis of the SERPINA1 gene and AAT protein by the specialized Center in Pavia [14].

## 3. Results

Out of 200 pulmonary patients screened, 141 were found to have alpha1-antitrypsin (AAT) levels less than 120 mg/dL and were subjected to isoelectric focusing for phenotyping of the most common variants and genotyping, while 41 patients presented mutations of the SERPINA1 gene. The mean age of the 41 patients was 56.6 ± 15.7 years old. The majority of these patients were non-smokers (51.0%) and former smokers (46%). Only one patient was an active smoker.

After the clinical evaluation, the high-resolution CT lung scan showed no findings in 12 patients (29%). Most of the patients with CT abnormalities showed evidence of emphysema (46% panlobular and 17% centrilobular) or other pathological findings. The most common clinical pictures were emphysema (44%) and COPD (39%); bronchiectasis was also found in six patients (14.6%), while pulmonary fibrosis in two patients (2.8%). Some patients had multiple pathological findings together (Table 1).

A total of 141 patients had serum levels of AAT < 120 mg/dL and were subjected to qualitative analysis. Among them, 36 showed an intermediate deficiency of AAT and five showed a severe deficiency of AAT. Subgroup analysis was performed for these 41 patients. Most of them showed an intermediate deficit of AAT with a mean value of 86.37 +/− 16.46 mg/dL, while the average serum AAT level in patients with the severe deficit was 49.50 +/− 31.02 mg/dL. Twelve patients had a serum alpha 1 antitrypsin value greater than 90 mg/dL, the maximum value was 119 mg/dL in two patients. No differences were found in other laboratory values as C reactive protein.

**Figure 1:**
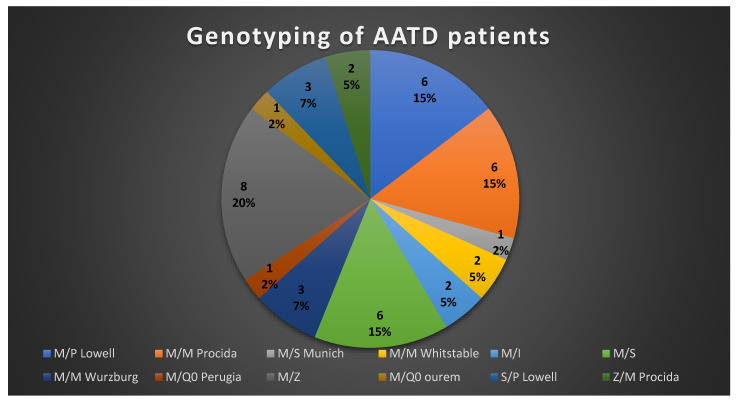
Genotyping of alpha-1 antitrypsin deficiency (AATD) patients. Samples from 41 patients with intermediate or low levels of AAT were subjected to genotyping, and the mutations identified are shown in this table.

Molecular analysis was conducted on 141 patients with AAT < 120 mg/dL. Of them, 41 were heterozygous or compound heterozygous for pathological mutations of the SERPINA1 gene, as reported in Figure 1. Commonly, the M allele is the most common allele and is associated with normal serum levels of AAT. The S allele produces moderate levels of AAT, and the Z allele produces very little AAT. The genotype PI*MS is associated with AAT levels about 80% of normal, while PI*MZ is associated with levels ≈50–70% of normal. The rare alleles determine a variable serum level depending on the variant of AAT.

Among 36 patients that showed an intermediate deficiency of AAT, 8 showed genotype PI*MZ and 6 showed PI*MS, while 22 showed other genotypes.

Six patients carried the mutation P_lowell_ [15]. These patients had emphysema and COPD. Six patients were identified with the mutation M_procida_ [16]; these patients were affected by COPD, obstructive sleep apnea syndrome, and fibromyalgia. One COPD patients had the mutation S_munich_ [17]. Three patients with emphysema had M_Wurzburg_ mutation [18]. Two patients had the mutation M_Whitestable_ [19]: one with pulmonary emphysema and the other with combined emphysema and pulmonary fibrosis pattern. Two patients were heterozygous for mutation I [20], and they had emphysema and bronchiectasis. One patient with COPD and lung cancer had the mutation Q0_ourem_ [21] and one with respiratory failure and emphysema had the mutation Q0_perugia_ [22] (Table 2).

Five patients had a severe AATD in serum (i.e., AAT levels <50 mg/dL).

## 4. Discussion

Alpha/1 antitrypsin deficiency is an autosomal codominant genetic condition that predisposes to the early onset of chronic pulmonary disease and liver disease. Even if AATD is one of the most widespread inherited diseases in Caucasian populations, identifying and diagnosing affected patients is still unsatisfactory, with only a minority of affected individuals being detected. Although WHO recommends that all patients with a diagnosis of COPD or adult-onset asthma should be tested [23], these recommendations are often disregarded [24]. Accurate and complete identification of the AATD geno/phenotype is clinically fundamental when deciding on potential treatment options for individual patients [25]. Methodological advances have facilitated the more widespread application of rapid, convenient, and cost-effective AATD tests, leading to an increase in the number of individual diagnosed with the disorder [26]. Laboratory diagnosis of AATD currently consists of serum biochemical analysis to evaluate protein deficiency and electrophoretic abnormalities, as well as genetic analysis to identify gene variants responsible for the protein deficiency [14].

The first step in diagnosing AATD is the quantitative test to determine the AAT concentration in plasma. It is a simple, inexpensive, and widely available test in most biochemical laboratories [27]. To interpret the result of an isolated quantitative determination, it must be considered that AAT is also an acute phase reactant. Therefore, infectious or inflammatory processes increase levels of AAT and give false normal or high values in patients with moderate deficiency. High levels of serum AAT during pregnancy and after the consumption of oral contraceptives have also been reported [28].

Indeed, the World Health Organization, the American Thoracic Society, and the European Respiratory Society recommend screening for AATD in patients with recurrent re-exacerbation pulmonary diseases and to test AAT level in a different moment from acute infection/inflammation. For the current study, 120 mg/dL accompanied by clinical evidence of lung disease was used as the decisional value to suspect AATD to address patients for further testing.

According to this selection, several SERPINA1 genotypes with different clinical features have been described. Nevertheless, the measurement of protein levels can identify patients with protein deficiency, but it cannot differentiate between the various AATD genetic subtypes. Therefore, molecular analysis of the AAT gene is the reference method to identify allelic variants.

Among 200 patients screened, 141 had AAT levels <120 mg/dL and underwent genetic testing. Of these, 36 (25.5%) were found to have an intermediate deficiency, and five (3.5%) were found to have severe AATD. This incidence is slightly higher than recent data based on the case-finding program in COPD in Spain, Ireland, and Argentina [29,30,31], but it is lower than those reported in the German population [32]. Therefore, since South Italy is a low AATD prevalence area, the frequencies of intermediate and severe AATD reported in the present study is higher than expected, thus supporting the correct approach in selecting patients for AATD testing.

The most prevalent SERPINA1 genotypes in the general Italian population were MS and MZ (5.8% and 1.5%, respectively) [16]. Accordingly, in the current study on patients with chronic pulmonary disease, MS and MZ mutations were the most common genotypes at 3.0% and 4.0% of the screened population and 14.6% and 19.0% of the population with reduced AAT, respectively [33].

PI*SS and PI*ZZ genotypes together are further rare pathological mutations that have a prevalence of 0.1% in the general population. Moreover, a study on the Italian population showed that the rare AATD variants displayed a different geographic distribution, peaking in some regions, for example in Sardinia. The authors also considered that the nomenclature of many rare AATD variants reflects their probable southern Italian origin (e.g., MProcida, MPalermo, Q0isola di Procida, Q0trastevere) [34]. Furthermore, concerning the distribution of rare mutations, another study focused on a North Italian area known to have a high incidence of AATD, showed that the prevalence of combined rare mutations was 0.5% [35].

As expected, we found a relative main difference in the distribution of AATD in the general population with a high incidence of AATD. In fact, in this study, in our cohort of 200 pulmonary patients, the prevalence in the overall patient population was 9.0%. Of course, the patients who were screened were selected patients with chronic pulmonary disease, and so this would explain the difference with the general population. A further selection analyzing patients with AAT levels <120 mg/dL found a combined global incidence of 20% (i.e., including MS, MZ and other rare alleles).

The main findings of our study are the high incidence of AATD in a selected cohort of patients tested in a specialist division of pulmonary disease as far as the identification of a considerable number of rare mutations causing AATD. Actually, there are only a few case reports on Italian cohorts [36,37], and this is the first report that looked for the prevalence of AATD in the geographic area of the city of Naples in Southern Italy.

From a clinical point of view, although the clinical condition most frequently associated with AATD is pulmonary emphysema [25], there are little data on the clinical presentation of rare mutations. In our study, patients with rare mutations showed frequently asthma and bronchiectasis as a main clinical feature.

Finally, our screening program enables us to diagnose a great number of rare mutations among patients with AATD, 27 rare alleles out of a total of 40 AAT alleles (67.5%), thus supporting the importance of an accurate molecular diagnosis that does not limit the testing to the S and Z variant. A recent paper showed a high prevalence of M_malton_ mutation in a small Italian cohort of patients admitted to the outpatient lung clinic in Parma [38]. In the present paper, we detected even eight different variants (M_procida_, P_lowell_, I, M_whitstable_, Q0_ourèm_, S_munich_, M_wurzburg_, Q0_peerugia_). This finding enables us to speculate about the genetic inhomogeneity of the Neapolitan population, which was historically submitted to migratory and conquest movements.

Further larger studies are needed to give improved information not only from an epidemiological point of view but also regarding the outcome of this clinical setting, in particular on our geographic area. For this objective, it should be fundamental to approach, as in our study, clinical data together with radiological findings and genetic screening to better understand the gene–environmental interactions and their influence on the clinical outcome.

## Figures and Tables

**Table 1:** Patients’ characteristics. Out of 200 pulmonary patients screened, 141 were found to have alpha1-antitrypsin (AAT) levels less than 120 mg/dL and were subjected to isoelectric focusing for phenotyping of the most common variants and genotyping, while 41 patients presented mutations of the SERPINA1 gene. The demographics and patient characteristics of these 41 patients are listed in this table. COPD = chronic obstructive pulmonary disease; CT = computed tomography.

Female/Male, *n* (%)	15/26 (36.5%/63.5%)
Age (years, mean ± SD)	56.6 ± 15.7
Smoking History	
Active Smokers, *n* (%)	1 (2.4%)
Former Smokers, *n* (%)	19 (46.3%)
Never Smokers, *n* (%)	21 (51.0%)
Clinical pattern	
Dyspnea, *n* (%)	4 (9.7%)
COPD, *n* (%)	16 (39.0%)
Bronchiectasis, *n* (%)	1 (2.4%)
Asthma, *n* (%)	3 (7.0%)
Emphysema, *n* (%)	18 (44%)
CT Features	
No findings, *n* (%)	12 (29.0%)
Panlobular Emphysema, *n* (%)	19 (46.0%)
Centrilobular Emphysema, *n* (%)	7 (17.0%)
Bronchiectasis, *n* (%)	6 (14.6%)
Fibrosis, *n* (%)	2 (2.8%)

**Table 2:** The M allele is the most common, and homozygosity (MM) is associated with normal alpha1-antitrypsin levels. The S, Z, and rare alleles are associated with varying degrees of AAT deficiency. The AAT variants are indicated with the corresponding mutation, the consequent anomaly, and the clinical data detected in our population.

Variant Name	Mutation	Consequences	Clinical Manifestation
Z	Glu 342 > Lys c. 1096G > A	Polymerization, decreased inhibitory activity; protein deficiency	Emphysema, COPD
S	Glu 264 > Val c. 863A > T	increased turnover; decreased inhibitory activity; mild protein deficiency	Emphysema, Asthma
P_Lowell_	Asp 256 > Val c. 839A > T	Polymerization, degraded in liver cells; protein deficiency	Emphysema, COPD
M_Procida_	Leu 41 > Pro c.194T > C	Intracellular proteolysis, decreased inhibitory activity; protein deficiency	COPD, Asthma
S_Munich_	Ser330Phe c. 1061C > T	Mild protein deficiency	COPD
M_Whitstable_	Intron mutation, 26 bp detection and 2 bp insertion in intron IV	Truncated protein, protein deficiency	Emphysema and fibrosis
I	Arg 39 > Cys c. 187C > T	Polymerization, slightly decreased inhibitory activity; mild protein deficiency	Emphysema, bronchiectasis
M_Wurzburg_	Pro369Ser c. 1177C > T	Degradation, protein deficiency	Emphysema
Q0_Perugia_	Val239→ DelG→STOP CODON241 V239GTG−delG > T ter241TGA	No detectable protein	Emphysema
Q0_ourem_	IVS1C + 1G→A exon5L352TTA, insT > Ter376TGA	No detectable protein	COPD, Lung cancer

## Data Availability

Data is contained within the article.
